# Antibiotic Prescribing in Dental Medicine—Best Practices for Successful Implementation

**DOI:** 10.3390/tropicalmed9020031

**Published:** 2024-01-26

**Authors:** Oana Săndulescu, Liliana Lucia Preoțescu, Adrian Streinu-Cercel, Gülşen Özkaya Şahin, Mihai Săndulescu

**Affiliations:** 1 Department of Infectious Diseases I, Faculty of Medicine, Carol Davila University of Medicine and Pharmacy, 050474 Bucharest, Romania; 2 National Institute for Infectious Diseases “Prof. Dr. Matei Balș”, 021105 Bucharest, Romania; 3 Department of Translational Medicine, Faculty of Medicine, Lund University, 223 62 Malmö, Sweden; 4 Department of Laboratory Medicine, Section of Clinical Microbiology, Region Skåne, 221 85 Lund, Sweden; 5 Department of Implant-Prosthetic Therapy, Faculty of Dentistry, Carol Davila University of Medicine and Pharmacy, 17-23 Calea Plevnei, 010221 Bucharest, Romania; mihai.sandulescu@umfcd.ro

**Keywords:** antimicrobial use, antibiotic resistance, rational use, antibiotics, dentistry

## Abstract

With rising rates of antimicrobial resistance throughout the world, it is time to revisit antibiotic prescribing policies and practices, and dentistry is an important area for focused intervention, as it accounts for up to 15% of all antimicrobial prescriptions. In this narrative review, we have analyzed the current state of the knowledge, attitudes, and practice regarding antimicrobial use among dental professionals, and we have identified a set of seven recurring themes that drive inappropriate antibiotic prescribing in dental medicine. These include: 1. Prescribing antibiotics to delay or avoid dental treatment. 2. Overlooking the 5Ds—dental treatment (source control), dental condition (indication), drug (antibiotic choice), dose, and duration. 3. Relying on education from the distant past and on previous experience. 4. The heterogeneity of (too many) guideline recommendations leads to confusion and over-prescribing. 5. Decreased access to guideline information in private practice. 6. Psychological factors such as pressure to prescribe, comfort prescribing and the weekend effect, and 7. Feeling removed from antimicrobial resistance and externalizing responsibility. Based on the existing knowledge, we propose a framework based on four key pillars for focused intervention: 1. Education. 2. Internalizing responsibility. 3. Recognizing recurring counter-productive practices, and 4. Addressing recurring counter-productive practices. This framework can be applied in different dental settings to ensure best practices for the successful implementation of rational antimicrobial prescribing.

## 1. Introduction

Antimicrobials have saved millions of lives since their discovery a century ago [1] and are part of the indispensable therapeutic armamentarium of modern medicine. However, antimicrobial use does not come without cost. Specifically, antimicrobials differ from other drugs in that they are societal drugs—their use by one individual has deeper repercussions for the larger society [2] through selecting for antimicrobial resistance (AMR).

Precisely for this reason, antimicrobial prescription is a shared responsibility, across all prescribers and all medical specialties; across all settings, from pharmacy to community to outpatient and inpatient settings; and, as defined by the One Health concept, across all relevant sectors beyond human health, including animal health and the environmental sectors. Thus, the One Health approach is vital to ensuring that unnecessary or inappropriate antimicrobial use is avoided across all relevant settings, in order to reduce AMR selection pressure on all levels. In this review, we will focus on antibiotic use in the human One Health sector, specifically in dentistry.

Dental practices account for 4% [3] to 15.6% [4] of all antibiotic prescriptions across medical specialties, with an increasing upward trend during the COVID-19 pandemic [4], possibly due to defensive prescribing in situations where dental consultations could not be provided under pandemic circumstances, restrictions, and the temporary de-prioritization of non-urgent medical care [5]. However, the pandemic lockdowns have long-since ended whereas this trend of increased antibiotic prescription in dentistry continues to persist [4,6].

Given the high share of antimicrobial prescriptions associated with dental practice, it is becoming increasingly important to understand the factors driving antibiotic prescription, and to see how these can be addressed to increase the appropriateness of antibiotic use. Efforts are needed to ensure that these prescriptions are both necessary and correct, and antimicrobial stewardship (AMS) can prove a helpful aid for clinical decision making when employed correctly. However, in order to develop an AMS program, it is essential to first understand the factors that influence correct and incorrect antibiotic use in each particular setting.

### 1.1. Antimicrobial Resistance—Different Burdens in Different Geographical Areas

The rates of AMR and AMR-associated burden differ by country. A recent report on the global burden of bacterial AMR in 2019 showed that 4.95 million deaths were associated with AMR, and that infections due to AMR represented the third leading cause of death worldwide, after ischemic heart disease and stroke [7]. The highest rates of AMR-associated deaths per 100,000 people were seen in sub-Saharan Africa (98.9), followed by South Asia (76.8), with a global estimate of 64 deaths per 100,000 people [7]. 

The three most important clinical syndromes accounting for the largest share of AMR-associated deaths worldwide included lower respiratory tract infections, bloodstream infections and intraabdominal infections [7]. This is not surprising, as the reported rates of AMR organisms are high for these clinical infections. Specifically, in a study from Iran that looked at the pathogens isolated from bronchoalveolar lavage, methicillin resistance was documented in 75% of *Staphylococcus aureus* isolates; vancomycin resistance in 27.3% of *Enterococcus* spp. isolates; carbapenem (imipenem) resistance in 40% of *Enterobacter* spp. isolates, 36.4% of *Pseudomonas aeruginosa* isolates, and 18.2% of *Acinetobacter baumannii* isolates; and gentamicin resistance in 40% of *Enterobacter* spp., 9.1% of *Pseudomonas aeruginosa*, and 45.5% of *Acinetobacter baumannii* isolates [8]. By comparison, an older study from Argentina reported the following resistance rates in bronchoalveolar lavage pathogens from 2001 to 2003: 67.4% methicillin resistance in *S. aureus*; 23.4% carbapenem (imipenem) resistance in *P. aeruginosa*, 0% in *K. pneumoniae*, and 48.4% in *Acinetobacter* spp.; and 33.0% amikacin resistance in *P. aeruginosa*, 26.0% in *K. pneumoniae*, and 76.8% in *Acinetobacter* spp. [9]. The rates of AMR in pathogens retrieved from bloodstream infections are also notoriously high, and particularly associated with poor outcomes. For example, in a study from Serbia, 77.8% of isolates of *P. aeruginosa* were resistant to imipenem, meropenem, and amikacin, while 100% of 195 tested isolates of *A. baumannii* were resistant to imipenem, meropenem, and amikacin, retaining susceptibility only to colistin (1.5% resistance) and, partially, to tigecycline (24.6% resistance) [10].

In high-income countries, the two leading pathogens contributing to the AMR burden were reported as *S. aureus* and *E. coli*, whereas in low- and middle-income countries from tropical regions such as sub-Saharan Africa, the leading pathogens implicated in AMR-associated deaths were reported to be *S. pneumoniae* and *K. pneumoniae* [7].

In the same reporting year (2019), a large percentage of AMR-associated deaths in the WHO European region occurred in the elderly, but several countries still reported a large number of neonatal and early life deaths due to AMR pathogens [11], similar to the profile seen in sub-Saharan Africa [12].

However, AMR respects no borders and has been spreading worldwide at an increasing pace over the past few decades, due to intensified international animal transport [13] on the one hand, and international human travel [14] on the other hand. A systematic review on travel-related antimicrobial resistance reported 30,060 AMR isolates from 26 identified bacterial species, which were more likely to be introduced into high-income countries from low- and middle-income countries [14]. The most important share of travel-associated resistance was seen for beta-lactams (35% of all resistance) and quinolones (31% of all resistance) [14], which represent the mainstay classes for the treatment of many, if not most, clinical infections.

### 1.2. Antimicrobial Resistance—The Ongoing Pandemic That Requires a One Health Approach

In his now-famous “Review on Antimicrobial Resistance” published in 2014, Jim O’Neill estimated that if all antimicrobials were compromised by resistance by 2050, we would be living in a world without antimicrobials, where infections untreatable because of AMR would cause an ominous 10 million deaths per year [15]. Despite this somber estimate, O’Neill’s review also provided a set of directions for concerted action to reduce inappropriate antimicrobial use and to preserve antimicrobial effectiveness, with the proposed solutions ranging from innovation to international governance, putting technological and scientific advances to good use, and investment in sanitation and health infrastructure where needed [15].

In the natural environment, wherever an interaction exists between bacteria and antibiotic substances, there is potential for AMR to emerge as an evolutionary self-preservation response. Naturally occurring resistance to penicillin was described in 1940 [16], even before the introduction of this antibiotic into clinical practice one year later, in 1941 [17]. With the increased use of antimicrobials in human and veterinary medicine, animal husbandry, and agriculture to name only a few applications, the selective pressure for bacteria to develop resistance has increased dramatically across the biosphere, and so have the rates of AMR organisms of clinical relevance [18].

Since antimicrobials are societal drugs, any type of use has a wider impact on the selection and propagation of AMR. For this reason, there is no one solution to AMR, and integrated approaches that span all relevant sectors should be considered and put into place in order to limit all avoidable antimicrobial use in a One Health approach. 

### 1.3. The Broader Consequences of Antimicrobial Use in Dental Medicine

From a One Health perspective, an essential interconnectedness exists between human, animal, and environmental health. For this reason, it is extremely important to consider the environmental dimension in the context of AMR. In this section, we focus on the impact of antimicrobial use in dental medicine on determining selective resistance pressure in the human host, and in the wider environment.

As mentioned above, any antibiotic use, whether appropriate or inappropriate, will lead to the exposure of bacteria to the antibiotic. On the patient level, this will be translated into exposure of the human microbiome to initially optimal but subsequently suboptimal antibiotic concentrations, as treatment is stopped and the antimicrobial drug is slowly eliminated from the body. This has the potential to induce resistance in the resident human flora, through mechanisms such as developing mutations in the antibiotic’s target site, producing enzymes that inactivate the drug, or the upregulation of the expression of efflux pumps, to name only a few. The bacteria that have thus acquired resistance can then be transmitted to other people, to animals, or excreted in the environment, or can cause an AMR-associated infection later on in the same individual.

Following the administration of antibiotics to humans, many of these drugs are excreted in an active form in urine, including members of important classes such as betalactams and fluoroquinolones, and some are excreted in their active form in feces, for example clindamycin. This will lead to the dispersion of antibiotics in sewage water and, subsequently, wastewater and surface water, a phenomenon which can also be accelerated by the inappropriate disposal of unused antibiotics. The presence of active antibiotic substances in environmental waters has been demonstrated for all major antibiotic classes, with a study from Poland identifying the presence of betalactams, macrolides, fluoroquinolones, tetracyclines, trimethoprim-sulfamethoxazole, and clindamycin in residual and surface waters [19].

In wastewater and environmental waters, abundant bacterial flora exists and, in the presence of antibiotics or antibiotic residues, the pressure to develop resistance occurs once again. Furthermore, as many different genera and species of bacteria and other microorganisms are present in wastewater and in environmental waters, this represents a perfect environment to facilitate transmission from one species to another of genetic material encoding for resistance, through horizontal gene transfer phenomena such as conjugation with the transmission of plasmids, transduction with the transfer of bacterial DNA by viral vectors such as bacteriophages, or even transformation, whereby bacteria can acquire free DNA from the environment [20].

Consequently, AMR bacteria have been identified in surface waters, with the study from Poland reporting that among *E. coli* environmental strains, 20.6% expressed an extended-spectrum betalactamase (ESBL) phenotype, 44.4% were multidrug resistant, and 1.6% were extensively drug resistant [19]. An extensive distribution of multidrug-resistant bacteria has also been reported for aquatic environmental samples from Germany [21], Romania [22], and many other countries.

Not only environmental water but also tap water has been shown to harbor AMR pathogens, with data from Nigeria showing that 42.9% of *Escherichia coli* isolates from community tap water harbored ESBL, a percentage higher than that seen in clinical isolates from the same region (39.1%), and comparable trends were found for the rates of metallo-betalactamase (28.6% vs. 33.3%) and AmpC-betalactamase (28.6% vs. 24.1%) [23]. When comparing isolates from tap water to clinical isolates of *Klebsiella pneumoniae*, rates of 26.3% vs. 36.5% were found for ESBL, 15.8% vs. 27.0% for MBL, and 15.8% vs. 22.2% for AmpC [23]. These data highlight the important bidirectional pressure between the environment and the clinical setting.

### 1.4. Aims of the Review

In this narrative review, we aimed to analyze the current state of the knowledge regarding antimicrobial use in dental medicine, to highlight essential areas of antibiotic prescription practices that could benefit from focused AMS interventions and to propose a framework that can be used to inform and develop best practices for the successful implementation of correct antimicrobial prescribing in dental practice.

## 2. Methods

### 2.1. Search Strategy; Inclusion and Exclusion Criteria

For the purpose of this narrative review, we have performed a search of PubMed, as the main medical publications database, using the following keywords: “antibiotic”, “dentists”, “knowledge”, and “attitude”, with the aim to identify articles meeting the criteria outlined below.

We selected for inclusion the articles that met all the following criteria: (a) original articles (b) from the past 10 years (c) reporting the main driving factors of antibiotic prescription among dental practitioners from different countries.

We excluded papers that met any of the following criteria: (a) review articles; (b) case reports; (c) conference abstracts, (d) full text not available in English. 

The search returned 97 results, of which 49 had been published within the past 10 years and were screened. Reference lists of the retrieved publications were also checked for potential additional relevant publications. Articles were further excluded if they did not contain information regarding antimicrobial prescription by dentists or if they did not report contributing factors, leaving a total of 28 articles for analysis (Table 1). These articles presented the results of questionnaire-based surveys, clinical audits, national registry studies, and semi-structured interviews with dental practitioners from the following countries: Norway (n = 1), Sweden (n = 1), France (n = 1), Spain (n = 1), Italy (n = 1), Bosnia and Herzegovina (n = 1), Cyprus (n = 1), Turkey (n = 1), Iran (n = 1), Jordan (n = 1), Lebanon (n = 1), Malaysia and Cambodia (n = 1), Dominican Republic (n = 1), Japan (n = 1), UK (n = 2), India (n = 2), USA (n = 2), Australia (n = 2), Saudi Arabia (n = 3), and Croatia (n = 6). 

### 2.2. Data Extraction

For each selected article, we extracted the following data: country or area where the study was performed; year in which the study was performed; type of study; type of dental personnel surveyed; type of antibiotic use surveyed. Furthermore, the factors identified as influencing antibiotic prescription were retrieved and grouped into 7 main categories, to illustrate commonly recurring themes that could eventually benefit from targeted intervention pathways aimed at improving prescribing practices.

## 3. Antimicrobial Use in Dental Medicine

Antimicrobials are used in dental medicine for the treatment of documented or suspected infections, and for the prophylaxis of local or systemic infections prior to a set of high-risk interventions [50]. However, the definition of “risk” is not entirely standardized across different medical and dental specialties, neither for patient profiles nor for types of procedures.

Despite the rather limited indications for antibiotics mentioned above, dental practices account for a non-negligible percentage of total antibiotic use in human medicine. Data from the UK Health Security Agency’s English surveillance programme for antimicrobial utilization and resistance (ESPAUR) report for 2021–2022 showed that, in England, antimicrobial prescription by dental practices represented 4% of the total consumption [3]. This percentage from dental medicine was equal to the total antibiotic use in community settings, and comparable to the 6% driven by hospital outpatient settings [3]. These numbers are even higher in other countries, as antibiotic use has paradoxically increased during the COVID-19 pandemic in many settings, and in dentistry in particular [4,6]. For example, 15.6% of antibiotic prescriptions between 2016 and 2021 in Norway were carried out by dental practitioners, amounting to almost 1 million prescriptions and 1.5 million defined daily doses of antibiotics in 2021 alone [4]. Furthermore, despite an overall decrease in antibiotic use in the country from 2016 to 2021 across all prescribing areas, in dentistry there was a significantly increasing trend [4], and the same was seen in Croatia [6]. Importantly, antimicrobials represent 72% of all medications prescribed by dentists [6].

These data show the important share of antimicrobial prescriptions that come from the dental field of practice, which highlights the importance of efforts to ensure that the prescriptions occurring in dentistry are both necessary and correct. This is of particular importance, since historical data suggest that most (81%) of the prophylactic antibiotic prescriptions prior to dental procedures were inappropriate, as reported in a study from 2011–2015 in the USA [44]. The same is true for most (81%) of the therapeutic antibiotic prescriptions for adult patients with acute dental conditions in 2012–2013 in the UK, which might also have been inappropriate [36]; more than half (65.6%) of the antibiotics prescribed by dentists in the UK study were given in situations where there was no actual evidence of infection [36]. 

## 4. Factors Influencing Antibiotic Prescribing Practices in Dental Medicine

It is essential to study and understand the factors that influence prescribing practices in dental medicine, but only a limited number of publications have focused on this topic. Furthermore, important differences can be seen across different settings, as many factors might be culturally driven.

A set of studies have assessed the knowledge, attitudes, and practices (KAP) regarding antibiotic prescription by dentists, and all have highlighted the need for more targeted education regarding the use of antibiotics by dental practitioners, and specifically for the following topics: relevant medical history to be considered when deciding whether to prescribe antibiotic prophylaxis [41,49], endodontic procedures that require antibiotic treatment [40,43] or antibiotic prophylaxis in at-risk patients [29,49], dental conditions that do and that do not require antibiotic treatment [41,49], AMR and AMS [40,43].

A study from the UK specifically looked at predictors for inappropriate antibiotic prescription in adult patients presenting to a general dentist for an acute dental condition in the absence of local or systemic signs of infection, from 2012 to 2013 [36]. This study found that the main drivers of antibiotic prescription were, in decreasing order of the odds of prescribing an antibiotic, the failure of a previous operative treatment (13.6-fold higher odds), insufficient time for operative treatment (10.2-fold higher odds), operative treatment refusal or contraindication (4.9-fold higher odds), patient request for antibiotics (3.7-fold higher odds), and the diagnosis of an acute periodontal condition (3.4-fold higher odds) [36]. However, these identified factors did not fully explain the prescribing practices, as significant between-dentist differences persisted in the statistical analysis even after adjusting for the patient, prescriber, and consultation attributes mentioned above [36], which suggests that in-depth interviews should probably be performed in order to better understand individual factors and to identify any recurring patterns or lines of thought.

**Figure 1:**
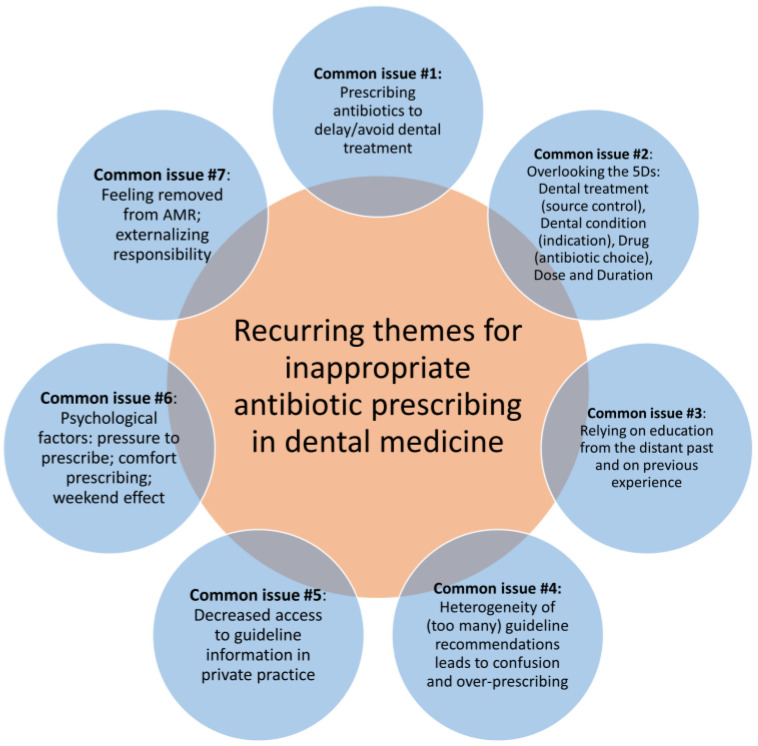
Recurring themes for inappropriate antibiotic prescribing in dental medicine. AMR, antimicrobial resistance.

Through this literature review, we have identified seven common recurring themes of inappropriate antibiotic prescribing in dental medicine. These are illustrated in Figure 1 and described in detail below.

### 4.1. Common Issue #1: Prescribing Antibiotics to Delay/Avoid Dental Treatment

In 70.6% of cases in the UK study referenced above, antibiotics were administered without source management [36]. A clinical audit performed among dental practitioners in Wales, UK, analyzed prescribing practices from 2012 to 2015 and reported comparable results to the study cited above, showing that 62.8% of patients who were prescribed antibiotics lacked signs of infection and that in 68.8% of cases no concomitant dental treatment was administered [35]. In a different study, 72.2% of respondents from four different countries (India, Malaysia, Saudi Arabia, and Cambodia) stated that they would prescribe antibiotics for patients who wished to delay elective treatment [42]. 

These last findings are particularly worrisome, since immediate source control can in many instances prevent antibiotic use altogether [51], while a lack of source control renders antibiotic treatment inefficient and unjustifiably prolongs treatment duration. Antibiotics should not be used in lieu of source control or to delay source control [51], except for the rare cases in which there is a direct contraindication for immediately performing the required dental procedure. 

Not having enough time to perform an emergency dental treatment is a major recurring theme in the reviewed publications [31,36,47]. In an interview with Australian general dentists, an interesting topic came up, specifically that, in larger clinics where front-desk staff manage appointments, the duration of emergency appointments scheduled with the patient’s routine practitioner might be as short as 15 min because “the patient just wants antibiotics” [47]. Emphasis should also be put on ensuring that reception staff are appropriately trained in order to discourage “antibiotic appointments” and to recognize that an emergency appointment might actually need a longer duration, in order for the appropriate dental treatment to be performed [47].

### 4.2. Common Issue #2: Overlooking the 5Ds: Dental Treatment (Source Control), Dental Condition (Indication), Drug (Antibiotic Choice), Dose, and Duration

The 5Ds of optimal antimicrobial prescription, as adapted for dentistry, represent the essential clinical decision pathway for each antibiotic prescription. These include a first step of deciding what dental treatment can be performed in order to ensure source control. This will be followed by establishing whether an antibiotic is or is not indicated for the patient’s dental condition, choosing the right antibiotic drug, and checking the correct dose and the shortest possible duration of administration [50]. 

Antimicrobials are inappropriately prescribed in many instances for conditions that are mainly inflammatory and not infectious, i.e., acute apical periodontitis, alveolar osteitis, or irreversible pulpitis, as cited from one study [35]. This is in line with data from Croatia and Bosnia and Herzegovina, where one third of those surveyed considered antimicrobials to be warranted in every oral inflammatory process [32]. This suggests the importance of stopping to think about the second D of rational antibiotic prescription: the question that should be asked is whether or not an antibiotic really is indicated for the patient’s dental condition. The answer might actually be “No” for many of the clinical situations encountered most often in dental practice. 

If an antibiotic is indicated, choosing the right drug might be more challenging in dentistry compared to other clinical indications, because of relatively few opportunities available to ensure an etiological diagnosis. In abscess-driven infections, the incision and drainage warranted for source control can also yield a cultivable pathological product, facilitating subsequent antimicrobial susceptibility testing. However, apart from these clinical situations, the exact etiology of most dental infections remains hard to establish and the treatment choice is based on the existing guideline evidence rather than on individual testing.

The clinical audit study from the UK also looked at the main reasons for deviating from the Scottish guidelines regarding the prescribed dose, frequency, or duration of antibiotic use; approximately one quarter (24.8%) of amoxicillin prescriptions were not compliant with the guidelines. The most frequent deviation from the guidelines was prolonged treatment duration (61.8% of cases of non-compliance to amoxicillin recommendations) [35].

Inappropriate treatment duration has indeed been consistently reported as a major issue throughout the literature in the field. A study from Lebanon by Mansour et al. reported the treatment duration as the main cause of non-conformity with evidence-practice guidelines, followed by the prescribed dose and, to a lesser extent, antibiotic choice (drug), and this pattern remained consistent across each individual prescribing indication and across treatment and prophylaxis options [39]. Specifically, a post hoc analysis of the data reported by Mansour et al. showed that the correct treatment duration was appropriately indicated by a median of only 3% (range 0% to 16.7%) of dental practitioners for prophylaxis, and by 37.5% (range: 0% to 45.7%) for treatment; the correct dose was appropriately indicated by only 16.7% of dental practitioners (range: 0% to 62.1%) for prophylaxis and by 26.7% (range: 0% to 78.3%) for treatment; the correct antibiotic choice was appropriately indicated by 33.3% of dental practitioners (range: 0% to 77%) for prophylaxis and by 60.6% (range: 32% to 87%) for treatment [39]. Furthermore, a significant correlation was found, in this post hoc analysis, between the appropriateness of the antibiotic choice and dose (r(20) = 0.8, *p* < 0.001), antibiotic choice and duration (r(20) = 0.6, *p* = 0.002), and duration and dose (r(20) = 0.5, *p* = 0.023) [39]. A worrisome finding from Australia is that the majority of the dentists interviewed chose the treatment duration not based on a guideline recommendation, but rather on the pack size, which led to longer treatment durations [47]. All these data suggest that particular emphasis should be put into the education of dental practitioners regarding appropriate doses and durations for antibiotic treatment; the data also suggest that improving one aspect of prescribing can have a global positive effect on overall prescribing practices.

Interestingly, dentists were more likely to appropriately recognize the clinical situations in which antibiotics are warranted than those where an antibiotic is not needed and should not be prescribed [26], which suggests that it is important to specifically deliver educational sessions focused on the clinical scenarios in which antibiotics are not warranted. 

### 4.3. Common Issue #3: Relying on Education from the Distant Past and on Previous Experience

In the study cited from Italy, dentists with more than 20 years of experience prescribed antibiotics more frequently, whereas younger dentists were more likely to be aware of the association between their prescribing patterns and AMR [27]. In a different study from Spain, dentists with more than 30 years of experience had 4.6-fold higher odds of suboptimal antibiotic prescribing practices [26]. Interestingly, in a study from Croatia, endodontists with less working experience (1–5 years) had the highest knowledge score regarding antibiotic use compared to their more experienced colleagues [29]. Dentists with more than 20 years of experience were also significantly more likely to prescribe wider-spectrum antibiotics (i.e., amoxicillin/clavulanate compared to amoxicillin alone) in a study from Saudi Arabia [41]. The same trend for better knowledge, better attitudes, and better performance was reported in Iran among endodontists who were younger and in proximity to their specialist training (younger specialists with less years in practice). 

Interestingly, the factor that influenced antibiotic prescribing behavior most frequently was “previous antibiotic experience”, as reported from Lebanon [39], suggesting that the prescribing patterns, particularly in more experienced practitioners, might be hard to change spontaneously, requiring targeted and potentially repeated educational activities. 

Furthermore, the knowledge acquired during undergraduate studies was mentioned as the main source of information by the majority of respondents to a study performed in the Dominican Republic, where only younger practitioners, aged 25 to 34 years old, said they also frequently consulted the scientific literature or specialized websites [46]. The same was true in a study from Japan, where dental school training was relied upon by 40% of respondents, regardless of the time span elapsed since graduation, including those who had graduated before the implementation of the first Japanese guidelines for the prevention of infective endocarditis; this source of information was closely followed by consulting with other dentists (40.6%), with medical doctors (46.4%), or consulting the guidelines (48.4%) [49]. The same pattern of “continuing to prescribe what they were taught at university” or asking for “advice from colleagues” instead of actively searching for the evidence base and the guideline recommendations, was also reported in interviews in Australia [47].

### 4.4. Common Issue #4: Heterogeneity of (Too Many) Guideline Recommendations Leads to Confusion and Over-Prescribing

It is encouraging to see that, in many settings, most dentists are open to receiving information regarding proper antimicrobial use [25,32,38,45,47] and AMR [25,38,47], and some go as far as mentioning that this “should be as important as getting your 60 h of CPD every two or three years” [47] and that if clinically relevant updates to non-dental guidelines are released, the information should also be sent to dentists [25]. Otherwise, antibiotic training is considered by dentists to be slightly out of the scope of dentistry, which makes it quite unlikely that they would find out about changes in other fields of practice [25,47], even though these changes can also have repercussions on their own practice. 

Furthermore, a study from Croatia highlighted the fact that the existence of multiple different guidelines for the prophylaxis of endocarditis, i.e., from the Croatian Cardiac Society, the American Heart Association, each with slightly different recommendations, is confusing to dentists’ practice [30], and the same was reported in the USA, where 68% of dentists and 64% of the surveyed medical doctors listed “conflicting guidelines and practices between professions” as an issue [45]. Email updates were indicated as the preferred means to receive regular information on antibiotic use by US dental professionals [45]. The risk of having multiple guidelines is very clearly described by a periodontologist, who states that “I find it more difficult than before, because in some cardiology protocols you now have to give antibiotics, in others you don’t have to. So, I tend to give them systematically when people are at risk of bacterial diffusion” [25].

### 4.5. Common Issue #5: Decreased Access to Guideline Information in Private Practice

The study from Lebanon reported a high rate of non-concordance with the guidelines, and highlighted the fact that working in a private clinic was significantly associated with the prescription of wider-spectrum antibiotics [41]. This is in line with data from Croatia [30] and France, where dental practitioners stated that those who also worked in a university hospital were familiar with the existing guidelines, but lower access to this type of information existed for those based in private practices only [25]. Rates of antibiotic overprescription were also higher in private compared to public emergency dental hospitals in Sweden, and so were the rates of antibiotic treatment not accompanied by dental treatment [24].

### 4.6. Common Issue #6: Psychological Factors—Pressure to Prescribe; Comfort Prescribing; and the Weekend Effect

The “pressure to prescribe” antibiotics upon patient request has been referenced in many of the identified studies [28,34,36,39,47]. For example, dentists interviewed from Australia stated that for certain demanding patients they do prescribe antibiotics on request, even when they know that there is no clinical indication [47]; moreover, 45% of dental practitioners from Lebanon have felt at some point this pressure to prescribe antibiotics at the patient’s request [39]. A significant percentage of dental practitioners surveyed in Croatia (43.4%) also cited patient request or expectation as being a reason to prescribe antibiotics [28]. In Turkey, general dentists were more likely to prescribe antibiotics at a patient’s request compared to specialist dentists or dental students, who had received training more recently [34].

In Italy, in the study by D’Ambrosio et al., the main reasons for mis-prescribing antibiotics, cited by dentists surveyed in 2021, were to avoid litigation (34.8%), unknown patient evolution in proximity to a weekend/holiday (23.5%), and non-compliant patients (23%) [27]. All of these factors are psychologically driven, and the pattern that prescribing antibiotics is somehow comforting for the patient, the prescriber, or both, has also been cited in other papers, as prescribing “[for patients to] psychologically feel that they’re being looked after” [47], “so that my patients trust that I am doing everything possible” [26] or “for [prescriber] peace of mind” [25], “to make extra sure for yourself and for the patient […] almost as a security blanket” [47]. The “weekend/holiday factor” is a very important determinant, as seen above [27], in which practitioners tend to prescribe antibiotics to avoid any unforeseen infectious complications over the weekend, with some practitioners going as far as stating that on Fridays they prescribe antibiotics to all patients, to avoid “the chance of a problem” occurring over the weekend, with the need for the patient to access emergency services [25]. However, the “weekend factor” is also seen in emergency dental services, where almost half (48.8%) of emergency visits received an antibiotic prescription, almost one third of visits (29.7%) received antibiotics alone without dental treatment, and antibiotics were significantly more frequently prescribed during Sundays and holidays (55.6% of visits) compared to working days (33.2% of visits) [31]. These are particularly alarming recurrent findings, suggesting that prescribing patterns might be more deeply rooted that previously considered.

### 4.7. Common Issue #7: Feeling Removed from AMR; Externalizing Responsibility

Almost all (98.9%) of the respondents from the study by D’Ambrosio et al. conducted in Italy stated that they were aware of the phenomenon of AMR, and 91.1% of them considered it to be “a growing phenomenon”, but less than half of them (42.9%) considered that their prescribing behavior was in any way associated with the development of AMR [27]. A study in Spain showed that the quality of antibiotic prescription was higher among dentists who perceived resistance as a public health problem [26]. This is a recurring theme, with recent qualitative interviews from France showing the same pattern, of a disconnect perceived by dentists and dental surgeons between their own prescribing habits and the phenomenon of antibiotic resistance [25]. Furthermore, even among those who acknowledge that resistance may be driven by their prescribing practice, narrower-spectrum antibiotics are not considered a concern, i.e., “just by prescribing amoxicillin” there should be no impact to public health [25], and neither does “a single course of antibiotics” have an impact [48]. 

Furthermore, 77.3% of endodontists from a study in India were not familiar with the concept of AMS or the World Health Organization (WHO)’s Access, Watch, Reserve (AWaRe) classification for antimicrobials [43], which highlights the importance of linking any information provided to dentists regarding antibiotics or AMR with information on rational prescribing practices. 

On a wider level, doctors, dentists, and veterinarians surveyed in Australia tended to externalize the responsibility of AMR and believed that the other professional categories played more important roles than their own in driving AMR [48]. This was also true in a study from Croatia and Bosnia and Herzegovina, where the surveyed dentists believed that excessive or unnecessary antibiotic use was less frequent in dental practice than in other branches of medicine [32]. Furthermore, in this study, most respondents recognized that unnecessary antibiotic use can be harmful (88.7%), but only half of them (56.5%) also recognized that appropriate antibiotic use can also contribute to AMR [32].

From a theoretical standpoint, being aware of the rising rates of AMR without understanding that these rates are actually driven by each of our prescriptions can hold the hypothetical risk of over-prescribing, and many of the reviewed studies highlight the fact that “personal responsibility for prudent antibiotic use should be increased” [28]. A recent viewpoint from Mendelson et al. highlights the importance of changing our discourse when talking about AMR. Specifically, the article conveys the following powerful message: “For far too long, we have exhorted people to join the ‘fight’ against antibiotic-resistant bacteria; our fight is not with bacteria, it’s with humans. It is us who overuse and misuse antibiotics, us who feed antibiotics to animals for food production, and us who pollute the environment through antibiotic manufacturing and other means” [18]. Planned education topics should also include activities that empower each prescriber to prevent the development of AMR by avoiding unnecessary antibiotic treatments. 

## 5. Discussion

In this review, we have identified the seven main recurring themes of inappropriate antibiotic prescription in dental medicine (Figure 1). In order to best address these, we have assessed the evidence from the field’s literature on interventions shown to improve dentist prescribing practices.

**Figure 2:**
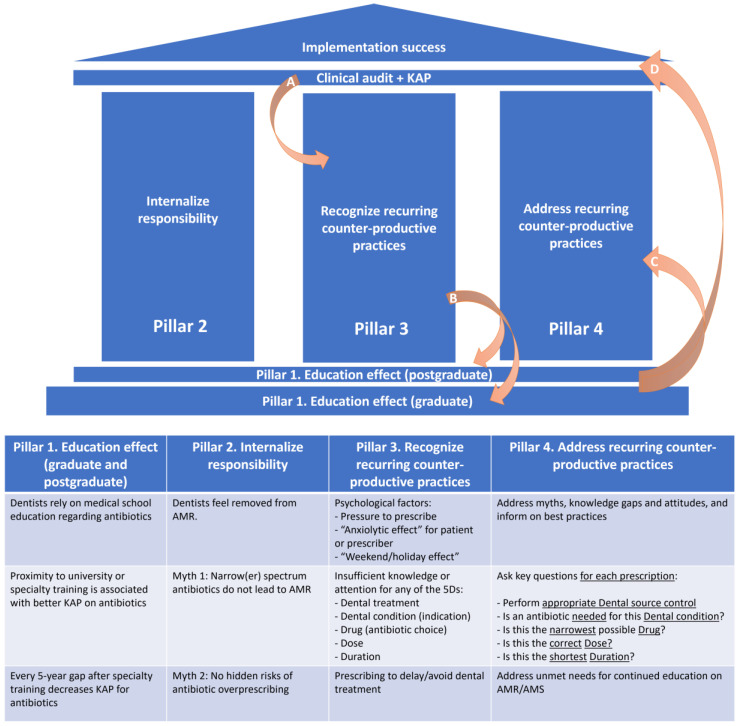
Proposed framework of key focused interventions to ensure the successful implementation of rational antibiotic prescribing practices in dental medicine. A. Clinical audits (1) and surveys of the knowledge, attitudes, and practices (2) regarding antibiotic use in dental practice represent important sources of information, since they can help in understanding the magnitude (1) of the phenomenon of inappropriate prescribing, and its main driving factors (2). B. Once recurring counterproductive antibiotic prescribing practices have been identified, postgraduate education should find the best training tools to address these by filling gaps in knowledge and addressing erroneous attitudes or perceptions. At this point, graduate education curricula should also be reassessed in order to determine whether revisions are needed to prevent further systematic prescribing errors from being introduced into dental practice. C. Once addressed through tailored educational solutions, D. implementation success follows. The cycle should be restarted at least every 5 years, to identify emerging issues. AMR: antimicrobial resistance; AMS: antimicrobial stewardship; KAP: knowledge, attitudes, and practices.

Furthermore, we have summarized these data into a proposed framework based on four key pillars of focused interventions, which interconnect to ensure the successful implementation of rational antibiotic prescribing practices in dental medicine (Figure 2). This framework can be used to develop tailored AMS interventions in different types of dental practices.

### 5.1. Pillar 1. Education Effects (Graduate and Postgraduate)

Medical practice in general, and dental practice in particular, are fields of work less dependent on automatization and more reliant on personal experience, on personal computing abilities, essentially on the ability to recognize a situation based on prior training and prior expertise, and to make informed decisions on how to best proceed. The more time elapses since the last training was received, the more one tends to rely on learned experiences rather than textbook information. However, this tendency to rely on previous experience can be dangerous if periodic re-attunement to correct and novel information is not provided.

A survey performed among medical, dental, and pharmacy students demonstrated a statistically significant education effect, with a decreasing likelihood of antibiotic use without prescription in parallel with increasing years of study, increasing knowledge levels regarding antibiotics, and, in particular, knowledge about how to prevent AMR [33]. This education effect was also shown to ensure better knowledge, better attitudes, and a better performance for prescribers in proximity to their specialty training (defined as a younger age or shorter clinical practice experience) among endodontists surveyed in Iran [37]. In a study from Turkey, students displayed higher knowledge scores regarding antibiotic use compared to dental practitioners, and so did dentists with less than 10 years of experience, compared to dentists with longer clinical experience [34]. 

Furthermore, an important effect has also been described for postgraduate education in Croatia, where this was associated with a positive impact towards appropriate antibiotic use, and specifically towards taking a moment to think whether or not an antibiotic prescription is warranted [28] (the second of the 5Ds).

A frequency of at least every 5 years for deploying postgraduate training on antibiotic use would probably represent the minimum educational standard [29]. A study performed in Colombia showed that a virtual learning environment for antibiotic prescription in dentistry significantly improved the awareness, attitudes, and intention to practice of dentists; however, 6 months after training, this positive effect was still present only for the awareness and intention to practice, while it had been lost for attitudes [52], suggesting that reinforcement might be required more frequently than the 5 years cited above [29].

Any type of information regarding antibiotic use should reinforce the implementation of the 5Ds for every single clinical decision. The following key questions should be asked before each antibiotic prescription: What dental treatment should I perform? Is an antibiotic needed for this dental condition (clinical indication)? Is this the narrowest possible antibiotic drug? Is this the correct dose? Is this the shortest duration? [50].

For successful implementation, the planned continuing education activities should be delivered periodically at the postgraduate level, but also incorporated into an updated university curriculum, as soon as the need for certain courses is identified. In particular, educational topics should also include activities that empower each prescriber to prevent the development of AMR by avoiding unnecessary antibiotic treatments.

### 5.2. Pillar 2. Internalize Responsibility

One of the main recurrent findings in the studied literature is that medical professionals feel removed from their responsibility for AMR, with resistance being seen as driven by external factors, or by other types of professionals, rather than by one’s own prescribing practice [48]. This is especially true for dental practitioners, who, in many instances, do not consider their prescribing to be connected to AMR [25], despite the fact that they actually contribute to quite a large share of the total antibiotic prescriptions in a country, as seen in Norway [4] or the UK [3]. This could be due to multiple contributing factors. 

First, there is insufficient knowledge regarding the link between AMR and any type of antibiotic prescription (appropriate or inappropriate, wide-spectrum or narrow-spectrum). This factor could in part be addressed by understanding the concept that antimicrobials are societal drugs, for which we all have a shared responsibly, and specifically by targeting the myth that narrow-spectrum antibiotics, or “a single prescription”, do not contribute to resistance.

Second, the real proportion of antibiotic prescription in dentistry is not acknowledged, i.e., dentists actually accounted for 15.6% of all antibiotic prescriptions in Norway [4] and antibiotics represent 72% of all types of medications prescribed by dentists in Croatia [6].

Third, the risks of over-prescribing should be fully recognized. Antimicrobial therapy comes with the risk of under- or over-prescribing. In cases where an antibiotic was under-prescribed, this will have immediate visible consequences on the patient, i.e., the flagrant progression of infection, which will provide a pro-antibiotic reinforcing feedback to the practitioner. This leads, in time, to over-prescribing based on previous experience. This could either mean prescribing when an antibiotic was not needed, or prescribing a wider spectrum than was actually needed. The issue here is that over-prescribing will have important consequences in terms of intra-patient AMR and wider, ecological repercussions in terms of AMR selection pressure, but will not actually have a directly palpable impact on the patient and will not be seen by either the patient or the prescriber. Except for rare toxicities, nothing of note will happen to the patient to show the practitioner that this was in fact a case of over-prescribing. If the antibiotic was actually not needed, the signs and symptoms will simply go away on their own or after the required dental procedure is performed. However, this natural course of improvement will be misattributed to the antibiotics, and because of the favorable patient evolution, the practitioner will continue to prescribe the same incorrect regimen to future patients with a similar clinical picture.

To address these issues, efforts should be directed towards internalizing responsibility for each individual antibiotic prescription. This could be achieved by further investing targeted efforts into providing insight on the environmental impact of antibiotic use, and this is closely linked with understanding the phenomenon of AMR and its ecological repercussions. Behavioral change interventions for improving antimicrobial use and for developing antimicrobial stewardship programs have been assessed in general medicine in many different settings, including primary care, outpatient services, inpatient services, private clinics, etc., however, the literature is extremely scarce for such interventions implemented in dentistry. Specifically, out of a total of 622 studies in human health retrieved for a systematic review that looked at behavioral change interventions for AMS, only two (0.3%) had been performed in a dental care setting [53]. This highlights the importance of including dental medicine in all types of interventions aimed at mitigating the ecological impact of antimicrobial use, alongside general medicine. 

Previous incorrect prescriptions should be addressed by periodic clinical audits and feedback, as well as case studies, where the appropriateness of antibiotic use is assessed and then explained to the practitioners, in order to break the vicious circle of prescribing based on previous (incorrect) experience. Furthermore, for case-based teaching activities, one of the main areas of focus should be on the clinical scenarios in which antibiotics are not needed and are commonly overprescribed, in order to address an important finding, i.e., that dentists more readily recognize the clinical situations which require an antibiotic prescription, while they often fail to recognize those than do not [26].

### 5.3. Pillars 3 and 4. Recognize and Address Recurring Counter-Productive Practices

A set of recurring counter-productive practices can be identified in the field’s literature. Each of these could be applicable to a higher or lower degree to each dental practice, depending on factors such as the type of dental specialty, type of patients, and individual- or community-based KAP. Each dental practice should assess their own KAP in order to determine what specific issues should be addressed [45]. 

For example, prescribing an antibiotic to delay or avoid source control through dental treatment is a recurring topic. This could be addressed by structural or educational interventions, depending on its root causes. If the issue at hand is actually that emergency appointments are too short to allow a dental treatment, then scheduling should be revised, and in order to do so it is essential to train the whole team (including front desk or receptionist staff, administrative personnel, and nurses) regarding the importance of source control to avoid unnecessary antibiotic treatment. This would ensure that scheduling allows enough time for dental treatment and also that patient request for unneeded antibiotics is discouraged from the very first point of contact with the dental clinic, i.e., from scheduling an appointment for a dental emergency.

Psychological factors include the patient-driven pressure to prescribe. This is a particular phenomenon seen more and more often in medicine in general, and it requires large amounts of time and energy and correct information on antibiotic prescribing in order to be correctly addressed. Whereas the pressure to prescribe is induced by the patient, a set of psychological factors are also specific to prescribers. These include the so-called “anxiolytic effect” of antibiotics, as well as the “weekend/holiday effect”, and both should be recognized and then fully addressed through all means mentioned above, i.e., (1) education and (2) the internalization of prescribing responsibility.

### 5.4. Implementation Success

Once all four pillars of the framework have been put into place, implementation success follows. However, most educational interventions are not long-lived and require periodic retraining. In order to understand how often retraining is needed and on what level—knowledge, attitudes, practices—clinical audits and feedback should be performed regularly. This will ensure that no major backload of inappropriate prescribing practices is cumulated, since any such buildup will potentially be more difficult to address later on.

This proposed framework is flexible and can easily be adapted to the specific needs of different dental practices in order to offer a tailored solution to improving antimicrobial practice.

### 5.5. Strengths and Limitations

This review comes with a set of limitations, derived from the nature of the articles identified by the literature search. While most of the studies included general dental practitioners along with those working in different dental specialties, quite a few of the studies included in an analysis (4/28) focused specifically on endodontics, which might to a certain extent polarize the results of the KAP analysis more towards antibiotic use in endodontics. Furthermore, 6 of the 28 studies identified were based in Croatia, a country that is overrepresented in the analysis.

However, this review also comes with a set of strengths. Specifically, approximately half (15/28) of the studies came from Europe, and the rest from other WHO regions, i.e., the Eastern Mediterranean region (6/28), the Western Pacific Region (3/38), the Americas (3/28), and the South East Asian Region (2/28), and all of the analyzed articles confirmed the same recurring patterns, which is reassuring as to the wider representativity of the findings highlighted here.

## 6. Conclusions

In conclusion, we have identified a set of seven recurring themes that drive inappropriate antibiotic prescribing in dental medicine, and we have created a framework based on four key pillars for focused intervention that can be applied in different dental settings to ensure best practices for the successful implementation of rational antimicrobial prescribing in dental medicine. These pillars emphasize the need to (1) promote education on correct antimicrobial prescriptions at the graduate and post-graduate level, (2) ensure the internalization of responsibility by understanding that each and every one of our antibiotic prescriptions will have a wider clinical and environmental effect, (3) recognize recurring counter-productive practices and (4) address them through continued education and tailored rational antimicrobial prescription models.

## Figures and Tables

**Table 1:** Summary of the main characteristics of the 28 articles included in the analysis.

No.	Country/Area of Study	Year When Study Was Performed	Type of Study	Type of Dental Personnel Surveyed	Type of Antibiotic use Surveyed	Reference
1.	Norway	2016–2021	National registry study investigating antibiotic prescription for 12 common antibiotics *	General dental practitioners, oral surgery, periodontics, endodontics, prosthetics, orthodontics, pedodontics, oral and maxillofacial radiology	P, T	Tousi et al. 2023 [4]
2.	Sweden	2016	KAP questionnaire and clinical audit	Dental practitioners working in two dental emergency clinics (public vs. private) (n = 1023)	P, T	Khalil et al. 2022 [24]
3.	France	2019–2020	Semi-structured interviews regarding the use of antibiotics and practitioner’s position regarding the antibiotic guidelines	Most were general dental practitioners, some were specialized in periodontology, implantology, or oral surgery (total n = 17)	P, T	Dormoy et al. 2021 [25]
4.	Spain	2021	Cross-sectional questionnaire study assessing prescribing quality	General dental practitioners, endodontics, periodontics, surgery, pedodontics, prosthodontics (total n = 878)	P, T	Rodríguez-Fernández et al. 2023 [26]
5.	Italy	2021	KAP questionnaire	Oral surgery, endodontics, orthodontics, pedodontics, periodontology, prosthetics/implantology (total n = 382)	P, T	D’Ambrosio et al. 2022 [27]
6.	Croatia	2018	KAP questionnaire	Dental practitioners (n = 230)	P, T	Farkaš et al. 2021 [28]
7.	Croatia	2020	KAP questionnaire assessing antibiotic prescription in endodontics	Most respondents worked in primary dental care, a few had dental specialization (total n = 657)	P, T	Simundic Munitic et al. 2021 [29]
8.	Croatia	Not specified, possibly 2019/2020/2021	KAP compliance with guidelines for IE prophylaxis	Any type of dental practitioner (n = 348)	P	Šutej et al. 2021 [30]
9.	Croatia	2015–2016	Retrospective cohort study on electronic medical records for antibiotic prescription	Dental practitioners working in emergency dental services in Zagreb (n = 20,879 clinical encounters)	P, T	Bjelovucic et al. 2019 [31]
10.	Croatia	2014–2018	Retrospective analysis of medications (including antibiotics) prescribed by dentists in the electronic registry of the Croatian Health Insurance Fund	n = 446,204 medication prescriptions (antimicrobials represented 72% of all prescriptions) by n = 2465 dentists	P, T	Šutej et al. 2021 [6]
11.	Croatia, Bosnia and Herzegovina	2017	KAP questionnaire	1/3 dental specialists, 1/3 dental residents, and 1/3 general dental practitioners (total n = 115)	P, T	Coric et al. 2020 [32]
12.	Cyprus	2020–2021	KAP questionnaire	University students; three groups: dentistry (n = 159), pharmacy (n = 101), and medicine (n = 54)	T	Baddal et al. 2022 [33]
13.	Turkey	2020	KAP questionnaire	Dentists and senior dental students (total n = 656)	P, T	Sirinoglu Capan et al. 2023 [34]
14.	UK (Wales)	2012–2015	Clinical audit of antimicrobial use	General dental practitioners (n = 279 dentists, each reviewed approximately 20 cases; n = 5760 clinical encounters)	P, T	Cope et al. 2016 [35]
15.	UK	2012–2013	Cross-sectional questionnaire-based study	General dental practitioners (n = 45)	T	Cope et al. 2016 [36]
16.	Iran	2019–2020	KAP questionnaire regarding the management of patients with chronic kidney diseases (including antibiotic use)	Endodontists (n = 100)	P	Arabpour et al. 2023 [37]
17.	Jordan	2021	Questionnaire-based study to evaluate preferred sources and awareness of available information and initiatives on rational prescribing practices	Dentists (n = 204)	P, T	Al-Taani et al. 2022 [38]
18.	Lebanon	2017	KAP cross-sectional telephone-based survey	General dentists, oral surgery, endodontics, implant surgery, pedodontics, orthodontics, restorative dentistry, prosthodontics, periodontics (n = 322)	P, T	Mansour et al. 2018 [39]
19.	Saudi Arabia	Not specified	Cross-sectional questionnaire study assessing attitudes towards antibiotic prescription during endodontic treatment	Dentists (n = 157)	T	Iqbal et al. 2015 [40]
20.	Saudi Arabia	2015	KAP questionnaire	General dental practitioners, specialists, etc. (total n = 373)	P, T	Halboub et al. 2016 [41]
21.	India, Malaysia, Saudi Arabia, Cambodia	Not specified	KAP questionnaire	47% general dentists while 53% had a specialist qualification (total n = 300)	P, T	Karobari et al. 2021 [42]
22.	India	2022	KAP questionnaire assessing antibiotic prescription during endodontic treatment	General dentists, endodontists, and other dental specialists (total n = 310)	T	Vengidesh et al. 2023 [43]
23.	USA	2011–2015	Retrospective cohort study. Dental visits linked to data from the national integrated health claims database	All—national health registry	P	Suda et al. 2015 [44]
24.	USA	Not specified. Probably before 2018	Knowledge, attitudes, and beliefs	Two groups: dentists (n = 84), medical doctors (n = 72)	P	McCarthy et al. 2020 [45]
25.	Dominican Republic	2016	KAP questionnaire regarding antibiotic prescriptions for pregnant and breastfeeding women	Dental practitioners attending a training course at the university (n = 98)	T	Aragoneses et al. 2021 [46]
26.	Australia	2018	Semi-structured interviews	General dentists (n = 15)	P, T	Teoh et al. 2019 [47]
27.	Australia	2016	KAP questionnaire focused on One Health use of antibiotics	Three groups: dentists (n = 380), medical doctors (n = 547), and veterinarians (n = 403)	P, T	Zhuo et al. 2018 [48]
28.	Japan	2016–2017	Knowledge and practice questionnaire	General dentists (most) with more than 20 years of experience (n = 345)	P	Nomura et al. 2018 [49]

* The antibiotics analyzed were doxycycline, oxytetracycline, tetracycline, amoxicillin, phenoxymethylpenicillin, amoxicillin/clavulanate, erythromycin, spiramycin, clarithromycin, azithromycin, clindamycin, and metronidazole. IE, infective endocarditis; KAP, knowledge, attitudes, and practice; P, prophylaxis; T, treatment.

## Data Availability

No new data were created or analyzed in this study. Data sharing is not applicable to this article.
